# Prevalence and risk factors for periprosthetic fracture in older recipients of total hip replacement: a cohort study

**DOI:** 10.1186/1471-2474-15-168

**Published:** 2014-05-22

**Authors:** Jeffrey N Katz, Elizabeth A Wright, Julian JZ Polaris, Mitchel B Harris, Elena Losina

**Affiliations:** 1 Orthopedic and Arthritis Center for Outcomes Research, Department of Orthopedic Surgery, Brigham and Women's Hospital, Harvard Medical School, Boston, USA; 2 Division of Rheumatology Brigham and Women’s Hospital, Harvard Medical School, Boston, USA

**Keywords:** Hip replacement, Periprosthetic fracture, Fracture, Implant, Risk factor, Epidemiology

## Abstract

**Background:**

The growing utilization of total joint replacement will increase the frequency of its complications, including periprosthetic fracture. The prevalence and risk factors of periprosthetic fracture require further study, particularly over the course of long-term follow-up. The objective of this study was to estimate the prevalence and risk factors for periprosthetic fractures occurring in recipients of total hip replacement.

**Methods:**

We identified Medicare beneficiaries who had elective primary total hip replacement (THR) for non-fracture diagnoses between July 1995 and June 1996. We followed them using Medicare Part A claims data through 2008. We used ICD-9 codes to identify periprosthetic femoral fractures occurring from 2006–2008. We used the incidence density method to calculate the annual incidence of these fractures and Cox proportional hazards models to identify risk factors for periprosthetic fracture. We also calculated the risk of hospitalization over the subsequent year.

**Results:**

Of 58,521 Medicare beneficiaries who had elective primary THR between July 1995 and June 1996, 32,463 (55%) survived until January 2006. Of these, 215 (0.7%) developed a periprosthetic femoral fracture between 2006 and 2008. The annual incidence of periprosthetic fracture among these individuals was 26 per 10,000 person-years. In the Cox model, a greater risk of periprosthetic fracture was associated with having had a total knee replacement (HR 1.82, 95% CI 1.30, 2.55) or a revision total hip replacement (HR1.40, 95% CI 0.95, 2.07) between the primary THR and 2006. Compared to those without fractures, THR recipients who sustained periprosthetic femoral fracture had three-fold higher risk of hospitalization in the subsequent year (89% vs. 27%, p < 0.0001).

**Conclusion:**

A decade after primary THR, periprosthetic fractures occur annually in 26 per 10,000 persons and are especially frequent in those with prior total knee or revision total hip replacements.

## Background

Total hip replacement (THR) is a highly effective treatment for advanced hip arthritis. It is an increasingly common procedure, with more than 300,000 primary THRs performed in the US every year [1]. As the number of patients with prosthetic joints in place increases, a growing number of individuals are at risk for implant-related complications, including periprosthetic fracture [2-5]. This particular complication is especially important and clinically relevant because it is typically costly, disabling and morbid. In fact, over 80% of periprosthetic fractures needed to be treated surgically in one large series [6].

It would be useful for patients who have had THR, as well as their providers and families, to understand the risk and subsequent clinical implications of sustaining a periprosthetic fracture. Prior estimates suggest that about 3.5% of THR recipients may experience a periprosthetic fracture over the first decade after THR [2]. Few studies have examined the prevalence of these fractures over longer-term follow-up, and there is relatively little information on risk factors for periprosthetic fracture. Limited evidence suggests an increase in risk due to older age [2], peptic ulcer [7], cardiovascular disease [7], and a loose implant [8,9].

The objective of this study was to estimate the incidence and risk factors for periprosthetic femoral fractures in a population-based cohort of patients ten to twelve years following primary THR. A secondary objective was to assess the effects of periprosthetic fracture on subsequent health care utilization by determining the one-year risk of hospital admission for THR recipients who developed periprosthetic femoral fractures as compared with recipients who did not sustain a fracture. We hypothesized that these fractures are associated with substantial resource utilization.

## Methods

### Sample

We identified Medicare beneficiaries who had elective primary THR, for non-fracture diagnoses, between July 1995 and June 1996. We followed this cohort in Medicare Part A claims data through 2008. The selection of this cohort has been described elsewhere [10,11]. We used ICD-9 diagnosis codes to identify periprosthetic femoral fractures for the period from 2006–2008. The ICD-9-CM code for periprosthetic fracture (996.44) was introduced in October, 2005, which is why we examine fractures starting in 2006. The ICD-9 code does not identify the side (right vs. left) of the fracture. We excluded patients with claims for periprosthetic fracture that included surgical procedure codes for the knee, tibia/fibula, humerus, radius/ulna, or shoulder in the absence of any procedure codes for the hip. We also identified all hospitalizations in the cohort of THR recipients occurring in 2006–2008. For this analysis we also excluded non-periprosthetic femoral fractures.

### Analyses

We used the incidence density method to calculate the annual incidence of periprosthetic femoral fractures. Time “at risk” was defined from the beginning of observation (January 2006) to one of the three events, whichever occurred first: 1) death, 2) periprosthetic fracture, or 3) end of the observation period (December 2008). Once the patient reached one of these endpoints, observation ceased.

We used multivariate Cox proportional hazards models to identify risk factors for periprosthetic fractures. Factors examined included sex, age at follow-up in 2006 (75–80 vs. >80), race (white vs. non-white), eligibility for Medicaid at time of primary THR (a proxy for low income), number of non-elective hospitalizations in the prior decade (a proxy for comorbidity, categorized as 0–2, 3–5, >5 hospitalizations), and having had a primary or revision hip or knee replacement after the index THR in 1995–96. Note that with regard to age, all cohort members were at least 75 years old in 2006 since they underwent THR as Medicare beneficiaries (and thus were at least 65 years old) in 1995–96.

In order to evaluate a prior observation that peptic ulcer and cardiovascular disease were associated with periprosthetic fracture [7,12], we also identified subjects who had prior admissions (from 1995–2005) with diagnostic codes for peptic ulcer disease (codes 531 through 535) and cardiovascular disease (codes 401 through 438).

We performed additional analyses to examine the consequences of these fractures for resource utilization over the subsequent year. For patients who had a periprosthetic femoral fracture in 2006 or 2007, we calculated the risk of hospitalization (to acute care, including acute rehabilitation facilities) over the year following the fracture. For comparison, we also calculated the one-year hospitalization risk for patients who did *not* have a femoral fracture in 2006 or 2007 from a randomly selected start date in 2006–2007. To identify the effect of periprosthetic fracture independent of age, sex and comorbidity, we ran a logistic regression with hospitalization as the dependent variable and periprosthetic fracture, as well as age, sex and Charlson comorbidity score as independent variables. We also examined the first-listed diagnosis on these admissions.

All analyses were done using SAS version 9.1.3. The study was approved by the Partners HealthCare Human Investigation Committee. Patient consent is not required to use administrative data provided proper procedure is followed. The investigators completed Data Use Agreements with the Centers for Medicare and Medicaid Services.

## Results

### Study sample

Of 58,521 Medicare beneficiaries who had elective primary THR between July 1995 and June 1996, 32,463 (55%) survived until January 2006. Their mean age was 82.5 and 67% of them were female (Table 1).

**Table 1:** **Demographic characteristics of Medicare recipients who had periprosthetic fracture or no fracture in 2006**–**2008 following primary THR in 1995**-**1996**

**Characteristic**	**Status in 2006-2008**	
	**Periprosthetic fracture (N** **=** **215)**	**No fracture (N** **=** **31,443)**
Age in 2006		
75-80 (%)	81 (38%)	12,548 (40%)
> 80 (%)	134 (62%)	18,895 (60%)
Sex		
Female (%)	139 (65%)	20,887 (66%)
Male (%)	76 (35%)	10,556 (34%)
Race		
White (%)	208 (98%)	29,556 (95%)
Non-white (%)*	5 (2%)	1,601 (5%)
Mean number of hospitalizations in 1995–2006 (range)	3.5 (0–20)	3.4 (0–43)
Mean number of hip or knee replacements after index THR and before 2006	0.49 (0–3)	0.38 (0–4)

*296 missing race.

### Incidence and risk factors

Between 2006 and 2008, 215 (0.7%) patients developed a periprosthetic proximal femoral fracture, for an annual incidence of 26 per 10,000 person-years.

The multivariate model identified having had a total knee replacement between the index THR and 2006 as a risk factor for periprosthetic fracture (HR 1.82, 9% CI 1.30, 2.55) as well as a revision total hip replacement between the primary THR and 2006 (HR 1.40, 95% CI 0.95, 2.07; Table 2). In the multivariate model, we did not observe an increased risk of periprosthetic fracture associated with cardiovascular disease (HR 0.87, 95% CI 0.62, 1.23) or with peptic ulcer disease (HR 0.98, 95% CI 0.62, 1.54).

**Table 2:** **Factors associated with risk of periprosthetic and non**-**periprosthetic femoral fracture in 2006**–**2008 among Medicare recipients who had THR in 1995**-**1996**

**Factor**	**Predictors of periprosthetic fracture**
	OR (95% CI)*
Age (in 2006):	
75-80	1.0
>80	1.26 (0.96, 1.67)
Sex:	
Male	1.13 (0.85, 1.50)
Female	1.0
Hospitalizations 1995-2006:	
0-2	1.0
3-5	0.94 (0.69, 1.29)
>5	1.06 (0.72, 1.57)
Hip or knee replacements after index THR and before 2006:	
Primary total hip replacement	
No	1.0
Yes	1.04 (0.75, 1.42)
Primary total knee replacement	
No	1.0
Yes	1.82 (1.30, 2.55)
Revision hip or knee replacement	
No	1.0
Yes	1.40 (0.95, 2.07)

*Adjusted for factors on table and Medicaid eligibility; race.

not analyzed because there were too few non-whites.

### Subsequent hospitalization

We calculated the risk of hospitalization over the next year for those cohort members who had periprosthetic fracture in 2006–2007 as well as for subjects who did not have femoral fracture for a year following a randomly selected date. Compared to those without fractures, THR recipients who sustained a periprosthetic femoral fracture had three-fold higher risk of at least one hospitalization in the subsequent year (89% vs. 27%, p < 0.0001). The risk of hospitalization associated with periprosthetic fracture persisted after adjustment for age, sex and comorbidity score (OR 23.0, 95% CI 15.0, 35.2). . Seventeen percent of the admissions among fracture patients were for medical reasons and the remainder for acute rehabilitation and other fracture related indications.

## Discussion

Total hip replacement is among the most successful medical advances of the twentieth century, relieving pain and improving functional status in approximately 90% of THR recipients [13]. Along with their new implants, however, patients who undergo THR take on the risks of several serious implant-related complications including dislocation, infection and periprosthetic fracture. The latter is perhaps the least well studied of these important complications and may be especially morbid, costly and disabling.

We evaluated the risk of periprosthetic fracture in a population-based study of Medicare recipients undergoing primary THR in 1995–1996. We estimated that the incidence of periprosthetic fracture among these individuals, who had THR about ten years earlier, was 26 per 10,000 person years. We observed that a prior knee replacements or revision THR were associated with greater risk of periprosthetic fracture, whereas typical risk factors for osteoporosis (e.g. older age, female sex) were not associated with the risk of these fractures. Periprosthetic fractures were associated with considerable subsequent health care resource utilization, with three-fold higher risk of subsequent hospitalization than experienced by THR recipients of the same age who did not have a femoral fracture.

Ours is the first US population-based study of periprosthetic fracture and the first study to our knowledge that focuses on the risk of these fractures many years after the hip replacement surgery. Thus our estimates capture the longer-term risk of living with a hip implant, as distinct from the perioperative risk of undergoing hip replacement surgery. Our work complements prior studies that have examined periprosthetic fractures in the Swedish [8,9] and Finnish [4] Hip Registers and large single center registries [7,14]. It is difficult to compare risks of fracture across these studies because of differences in patient age and the timing of the follow-up window in relation to the primary THR. Our findings suggest that the higher earlier risk of these fractures in the perioperative period diminishes considerably over long term follow up. Our findings show that in this older age group, men and women have similar risk of periprosthetic fracture, suggesting that clinicians should retain a high index of suspicion of periprosthetic fracture in an older patient with a compatible history even in the absence of traditional osteoporotic risk factors. The increased risk associated with concomitant knee replacement and revision hip replacement may relate to the mechanical stiffness associated with multiple implants, especially on the same limb. Singh and colleagues found that a history of peptic ulcer disease and of heart disease were risk factors for periprosthetic fracture. We were not able to confirm this observation, although our comorbidity data were derived from inpatient claims, which are likely insensitive indicators, particularly of peptic ulcer disease.

As with any analysis of claims data, our study was limited by potential misclassification in the outcome (periprosthetic fracture). Also, we were unable to perform analyses for periprosthetic fracture for years prior to 2006, when the codes for these fractures were created. We were unable to elicit several clinical details that might influence risk of periprosthetic fracture such as whether the implant was cemented or uncemented and we did not have access to pharmacy data, which might have shed light on the role of medications that increase risk of falls and treatments for osteoporosis.

## Conclusion

We conclude that periprosthetic fracture occurs in about one in 400 THR recipients per year once these patients are 10 years out from their hip replacement. These data will help clinicians as they portray to patients and their families the long-term concerns associated with living with a hip implant. The message is that periprosthetic fractures are relatively rare, though more frequent in patients with multiple implants. Further, these fractures are typically associated with the need for considerable subsequent medical care, as they are accompanied by a much greater risk hospitalization in the subsequent year than experienced by THR recipients who did not have hip fracture.

## Competing interests

The authors declare they have no competing interests.

## Authors’ contributions

JNK conceived of the study, oversaw the analyses, led discussions of the approach and the findings, drafted sections of the manuscript, edited the entire manuscript carefully. EAW performed the statistical analyses, drafted select sections of the manuscript, and edited the manuscript carefully. JJZP drafted most of the manuscript, participated in all discussions of approach and findings and edited carefully. MBH participated in discussions of the conceptual and clinical underpinnings of the project and of the findings and carefully edited the manuscript. EL participated in conception of the project, in providing senior level methodological consultation, discussing the findings and their implications and carefully editing the manuscript. All authors read and approved the final manuscript.

## Pre-publication history

The pre-publication history for this paper can be accessed here:

http://www.biomedcentral.com/1471-2474/15/168/prepub
